# Responses of Fungi Maggot (*Bradysia impatiens* Johannsen) to Allyl Isothiocyanate and High CO_2_

**DOI:** 10.3389/fphys.2022.879401

**Published:** 2022-05-05

**Authors:** Yu-Ping Gou, Peter Quandahor, Liang Mao, Chun-Chun Li, Jing-Jiang Zhou, Chang-Zhong Liu

**Affiliations:** ^1^ College of Plant Protection, Gansu Agricultural University/Biocontrol Engineering Laboratory of Crop Diseases and Pests of Gansu Province, Lanzhou, China; ^2^ CSIR—Savanna Agricultural Research Institute, Tamale, Ghana; ^3^ Forestry and Grassland Bureau of Lintao County, Dingxi, China

**Keywords:** *Bradysia impatiens* Johannsen, allyl isothiocyanate, sub-lethal effects, high CO_2_, detoxification enzyme activity, antioxidant enzyme activity

## Abstract

Botanical pesticide is highly recommended for integrated pest management (IPM), due to its merits such as environmental friendliness, safe to non-target organisms, operators, animals, and food consumers. The experiment was conducted to determine the lethal and sub-lethal effects of allyl isothiocyanate (AITC) on eggs, third instar larvae, pupae, and females and males of *Bradysia impatiens* Johannsen (*B. impatiens*). Different concentrations of AITC under ambient CO_2_ by the conical flask sealed fumigation method were used for the experiment. The results showed that there was a significant linear relationship between different concentrations of AITC and the toxicity regression equation of *B. impatiens*. The sub-lethal concentrations of AITC had significant effects on the larval stage, pupal stage, pupation rate, pupal weight, adult emergence rate, and oviposition. The pupation rate, pupal weight, and adult emergency rate were significantly (*p* < 0.05) affected by AITC fumigation. The pupation rate was the lowest after fumigation treatment of AITC at LC_50_ (36.67%), followed by LC_25_ (41.94%), compared with the CK (81.39%). Female longevity was significantly (*p* < 0.05) shortened by fumigation at LC_25_ (1.75 d) and LC_50_ (1.64 d), compared with that of CK (2.94 d). Male longevity was shorter at LC_25_ (1.56 d) than at LC_50_ (1.25 d) and had no significant difference between these two treatments. The fumigation efficiency of AITC was significantly increased under high CO_2_ condition. Furthermore, detoxification enzyme activities and antioxidant enzyme activities were accumulated under high CO_2_ condition. The fumigation method in the application of AITC can be useful in areas where *B. impatiens* is a major concern.

## 1 Introduction

*Bradysia impatiens* Johannsen is an economic important pest globally, which survives on mushrooms, chives, ornamental plants, and humus (Johannsen, 1912; Menzel et al., 2003; Santos et al., 2012; Gou et al., 2019). The larvae feed on the roots, stems, leaves, flowers, and even the whole seedling of host plants (Gou et al., 2020a; Gou et al., 2020b). *B. impatiens* was first reported on mushrooms in China (Shen et al., 2009). Other host plants of this pest include chive (*Allium tuberosum*) (Gou et al., 2015), onion (*A. fistulosum*) (Gou et al., 2015), lily (*Lilium brownie*) (Gou et al., 2015), carrot (*Daucus carota*) (Arimoto et al., 2018), poinsettia (*Euphorbia pulcherrima*) (Cheng et al., 2018), butterfly orchid (*Phalaenopsis aphrodite*) (Cheng et al., 2018), strawberry (*Fragaria ananassa*) (Sueyoshi and Yoshimatsu, 2019), and eucalyptus (*Eucalyptus robusta*). *B. impatiens* is an agricultural and forestry pest distributed in areas such as the United States, South Africa, Japan, the Netherlands, Brazil, Hawaiian Islands, the United Kingdom, Russia and other countries (Menzel et al., 2003; Gou et al., 2020b).

Botanical pesticide is highly recommended for integrated pest management (IPM) due to its merits of environmental friendliness, safe to non-target organisms, operators, and animal and food consumers. The development of the botanical control method has been proposed as one of the most important strategies for pest management on fruits, vegetables, and ornamental plants (Zhang et al., 2015; Cheng et al., 2018; Lu et al., 2020). AITC, commonly known as horseradish, is a volatile and aliphatic sulfur-containing compound naturally occurring in plants from the family of Cruciferae, such as: horseradish (*Armoracia rusticana*), mustard (*Brassica nigra*), cabbage (*Brassica oleracea*), and wasabi (*Wasabia japonica*) (Mayton et al., 1996; Wu et al., 2009; Williams et al., 2015; Li Y et al., 2018). AITC could be rapidly adsorbed and degraded in soil with a low risk of persistence (Ren et al., 2018); thus, it has been considered a potential botanical pesticide. It has been effectively used to control soil-borne fungi (Yim et al., 2016), plant pathogen (Smolinska et al., 2003; Troncoso et al., 2005; Ugolini et al., 2014), nematodes (Brolsma et al., 2014), weeds (Bangarwa and Norsworthy, 2014; Devkota and Norsworthy, 2014; Matteo et al., 2017), and pests (Wu H. et al., 2014; Deguenon et al., 2019; Zhang et al., 2020). Some storage pest species, including *Sitophilus oryzae* (Worfel et al., 1997), *Sitophilus zeamais* (Mansour et al., 2012), *Callosobruchus maculatus* (Zhang et al., 2016), *Tribolium confusum* (Li et al., 2011), and *Plodia interpunctella* (Liang et al., 2013), are particularly sensitive to AITC and have achieved remarkable fumigation effect. AITC also has good efficacy against adults of *B. odoriphaga* (Shi et al., 2017).

An increase in atmospheric carbon dioxide (CO_2_) concentration is predicted to continually increase from the current 400 ppm to between 750 and 1,300 ppm by the end of this century (Erda et al., 2005; Li et al., 2020). CO_2_ is an important regulator of respiration, and the spiracle of insects are kept open permanently under high concentrations of CO_2_ (higher than 10–20%) (Miller, 1974). Accordingly, they are likely to absorb more fumigants when their spiracle is open permanently. A mixture of fumigants and high concentrations of CO_2_ enhances toxicity to insects (Miller, 1974; Haritos et al., 2006). Several positive effects on various fumigants have been investigated, including methyl bromide, ethanedinitrile (Ramadan et al., 2021), ethyl formate (Haritos et al., 2006; Damcevski et al., 2010), and the essential oils from *Perilla frutescens* (L.) Britt. (Lamiaceae) (Ye et al., 2015), showing that the control efficacy against the targeted pests had been apparently strengthened under high concentrations of CO_2_. In general, there is a balance between the generation of reactive oxygen species (ROS) and their scavenging. However, when exposed to environmental stress, the balance is disrupted. Insecticidal stress causes an increase in the production of ROS, which causes oxidative damage (Lalouette et al., 2011). Excess ROS causes lipid peroxidation (LPO) and disrupts cell membrane fluidity, leading to cell lesions. The degree of membrane LPO can be determined indirectly by measuring the concentration of malondialdehyde (MDA) (Meng et al., 2009). Organisms have evolved complex adaptation-related mechanisms for eliminating ROS, such as molecular antioxidants and antioxidative enzymes (Joanisse and Storey, 1998), to maintain homeostasis and prevent ROS damage. Superoxide dismutase (SOD), catalase (CAT), peroxidase (POD), and glutathione-S-transferases (GSTs) are the most important components for protecting cells and maintaining homeostasis in various stress conditions by scavenging ROS (Felton and Summers, 1995). Numerous studies have used antioxidant responses to thermal stress as indicators of important physiological adaptation processes in insects (Felton and Summers, 1995). Detoxification enzymes in the insect body such as the GSTs, CarE, and cytochrome-b5 (Cyt-b5) are other important factors (Li et al., 2021; Liang et al., 2017) that decrease the effectiveness of insecticides by changing the metabolism (Xiao et al., 2009; Su et al., 2012).

*Bradysia impatiens* is considered an economic important pest due to its ability to inhibit the production of a wide range of agricultural crops. Consequently, wide ranges of synthetic insecticides are continuously used for its management in China. This type of strategy has been found to seriously increase environmental contamination and insecticide resistance and endangers the health of farm operators, animals, and food consumers. Botanical pesticides are considered safe in pest control because they have low or no toxic residue, making them safe to people and the environment. Thus, developing botanical control methods for the management of *B. impatiens* is paramount for crop production. This research is based on the hypothesis that the application of AITC and/or CO_2_ combination affects the performance of *B. impatiens*. The study is, therefore, conducted to determine the lethal and sub-lethal effects of allyl isothiocyanate (AITC) on eggs, third instar larvae, pupae, and females and males of *B. impatiens* Johannsen (*B. impatiens*).

## 2 Materials and Methods

### 2.1 Experimental Plant

The chive cultivar “pingjiu No. 2,” which is susceptible to *B. impatiens* (Gou et al., 2015), was planted in the experimental field of Gansu Agricultural University for 3 years. All necessary agronomic practices such as watering, stubble cutting, and farm manure application were carried out regularly, without chemical spraying.

### 2.2 Tested Insect

Larvae of *B. impatiens* were collected from chive plants in a greenhouse of the Pan’an town (34° 45′22′ N, 105° 7′2 ′E), Gansu County, Tianshui, China, in April 2019. The individuals of the laboratory population were reared on a self-developed artificial diet (Gou et al., 2020c). The eggs were collected and placed in transparent plastic rearing containers (upper diameter × lower diameter × height = 12 cm × 8 cm × 8 cm). The larvae were fed on chive rhizomes for three constant generations in a light growth chamber under (25 ± 1)°C temperature, 65–70% relative humidity (RH), and 16 L h: 8D h photoperiod. The eggs, third larvae, pupae, and newly emerged females and males were randomly selected for the analyses.

### 2.3. Chemicals

Technical grade AITC (active ingredient >98%) was purchased from Sigma, the United States of America GSTs, CarE, Cyt-b5, SOD, CAT, and POD assay kits were purchased from Shanghai Preferred Biotechnology, China.

### 2.4 Toxicity Process

#### 2.4.1 Preparation of Allyl Isothiocyanate Solution

To achieve the mother liquor containing 9.5% AITC, technical grade AITC was dissolved in soybean oil with a volume ratio of 1: 9 V/V (Santos et al., 2011; Paes et al., 2012) and stored at 4°C in dark (maintain chemical activity). Soybean oil did not affect the toxicity tests on *B. impatiens* that was used as the solvent to minimize the volatilization rate of AITC, particularly when used at low quantities (Paes et al., 2012). Thus, preliminary experiments were conducted, and final concentrations of AITC including 0.0, 3.0, 6.0, 9.0, 12.0, and 15.0 μL/L were tested.

#### 2.4.2 Fumigation Test

The experiment was carried out in the fume hood of the laboratory under ambient CO_2_ by the conical flask sealed fumigation method (Ren et al., 2018). A count of 100 eggs (1 day old), third larvae, pupae (1 day old), and unmated females and males were collected each and then placed in corresponding flasks (500 ml). Each flask contained fresh chive rhizomes (2-cm length) and had wet absorbent cotton with two filter papers (9-cm diameter) at the bottom. Aliquots of the AITC test solution (30 μL) were applied to the filter paper strips (1 cm × 6 cm) plugged at the top of the flask and then sealed with plug and parafilm. The untreated control flask had only soybean oil (30 μL). Three replicates were set up for each treatment. All flasks were incubated 24 h in the light growth chamber at (25 ± 1)°C, 65%–70% RH, and 16: 8 (L: D). The adults and larvae were considered dead with no observable motion when gently touched with a soft brush. The pupae were recorded for 5 d to observe the adult emergence number. The egg hatching numbers were recorded for 7d.

### 2.5 Effects of Sub-lethal Concentrations of Allyl Isothiocyanate on Growth Parameters of *B. Impatiens*

The AITC sub-lethal concentrations (LC_25_ and LC_50_) against third larvae obtained by fumigation were used to determine the growth parameters of *B. impatiens*. The solvent soybean oil without AITC was used as the control. Each treatment contained 100 third larvae with three replications. After 24 h treatment, 60 surviving larvae were transferred into three petri dishes (12-cm, 20 individuals in one dish) containing moistened filter papers and maintained in a growth chamber at 25°C, 65%–70% RH, and 16L: 8D. Water was added along the edge of the filter paper every day to maintain appropriate humidity and timely supplemented with fresh chive rhizomes without chemicals. The survivals and instars were recorded. The pupae were moved to petri dishes (35-mm, containing moistened filter paper) one by one and weighed within 48 h. We weighed the pupae with a single head using a millionth scale. Twenty heads were collected for each treatment and repeated three times. Meanwhile, the survival of pupae was monitored daily. Once an adult emerged, unmated males and females (1:1) were transferred into transparent plastic containers (as described earlier). Each container contained absorbent cottons and filter papers; the male and female longevity and the number of eggs laid by each female were recorded daily. Ten pairs were considered a group, one group was considered a replicate, and three replicates were used for the female oviposition and adult longevity monitoring.

The pupation rate (%) was calculated using the formula [(total number of collected pupae/total number of fourth instar larvae) × 100]. The emergence rate (%) was calculated using the formula [(total number of emerged adults/total number of collected pupae) × 100].

### 2.6 High CO_2_ and/or LC_25_ Allyl Isothiocyanate Fumigation Stress

To assess the effect of elevated CO_2_ on AITC fumigation efficacy against *B. impatiens* larvae, 100 heads third larvae of *B. impatiens* were placed in one conical flask (500 ml) with three replicates. The experiment was carried out with LC_25_ concentration of AITC as described in the previous paragraph. In addition to the AITC fumigation + high CO_2_ (AF + HC, under high CO_2_ concentration about 800 ppm with AF stress), the flasks containing the larvae were connected to a high-pressure CO_2_ cylinder via plastic tubing. CO_2_ was delivered to the flasks for 10 s at 50 kPa (about 800 ppm) (Wang et al., 2018). The flasks were immediately tightly sealed with plug and parafilm. Larvae without any treatment (CK, under ambient CO_2_ concentration about 400 ppm without AF stress), larvae treated only with AITC fumigation (AF, under ambient CO_2_ concentration about 400 ppm with AF stress), and larvae treated with only high CO_2_ (HC, under high CO_2_ concentration about 800 ppm without AF stress) were all placed at the same time. At 24, 48, and 72 h after treatment, the larvae were recovered under ambient air for 2 h and mortality was examined. The larvae were considered dead if they were immobile after being gently stimulated by a soft brush.

### 2.7 Enzyme Activity Assay

#### 2.7.1 Preparation of Enzyme Sources and Determination and Calculation of Enzyme Activity

Third instar larvae of *B. impatiens* were collected from four treatments (CK, HC, AF, and AF + HC), washed with phosphate buffer solution (PBS, pH 7.0) containing 1.0 mmol/L ethylene diamide tetra acetic acid (EDTA), and then kept in a 1.5-ml centrifuge tube. Each tube contained 20 heads (about 0.05 g) with three replications and marked. Each sample was frozen in liquid nitrogen for 15 min and then homogenized in ice in 1.5 ml of 0.1 M PBS with a high-speed tissue grinder (TIANGEN). The supernatants were collected after the centrifugation of homogenates at 12,000 × g for 15 min at 4°C. The resultant supernatant was stored at −80°C and used as an enzyme source. We detected three detoxification enzyme activities, such as glutathione-S-transferase (GSTs), carboxylesterase (CarE), and cytochrome-b5 (Cyt-b5); three antioxidant enzyme activities, such as superoxide dismutase (SOD), catalase (CAT), and peroxidase (POD). The methods of determination and calculation for each enzyme were strictly referred to the instructions of the kit (Shanghai Preferred Biotechnology, China).

### 2.8 Statistical Analysis

The experimental data were statistically analyzed using SPSS23.0 software. The toxicity regression equation and the values of LC_25_ and LC_50_ were calculated by the Probit module (probability unit regression) (Wu H. H. et al., 2014). Differences among the treatments were subjected to Tukey’s test (*p < 0.05*). GraphPad Prism 6.0 software was used for plotting graphs.

## 3 Results

### 3.1 Toxicity of Allyl Isothiocyanate to *B. Impatiens*

The bioassay result indicated that AITC showed significant (*p* < 0.05) insecticidal effect on *B. impatiens*. After fumigation in the conical flask for 24 h, the values of LC_50_ against the female and male adults were 4.79 and 5.47 μL/L, respectively, which were significantly lower than those of the eggs (10.23 μL/L, *χ* ^2^ = 1.30, *p* = 0.71), pupae (10.40 μL/L, *χ* ^2^ = 1.92, *p* = 0.58), and third larvae (11.27 μL/L, *χ* ^2^ = 1.55, *p* = 3.38) (Table 1).

**TABLE 1:** Toxicity of the AITC fumigation method to substages of *B. impatiens*.

Substage	Toxicity regression equation	*χ* ^2^ (Df)	*p*-value	LC_25_ (μL L^−1^) (95%confidence limit)	LC_50_ (μL L^−1^) (95%confidence limit)
Eggs	*y* = −1.14 + 1.132*x*	1.39 (99)	0.71	2.60 (0.692–4.101)	10.23 (7.619–16.585)
3rd larvae	*y* = −1.41 + 1.382*x*	1.55 (99)	0.67	3.38 (1.583–4.739)	10.40 (8.161–14.968)
Pupae	*y* = −2.04 + 1.939*x*	1.92 (99)	0.58	5.06 (2.849–6.661)	11.27 (8.579–17.197)
Female	*y* = −2.40 + 3.527*x*	1.35 (99)	0.94	3.08 (0.622–4.677)	4.79 (2.067–6.851)
Male	*y* = −2.34 + 3.175*x*	1.38 (99)	0.81	3.35 (0.421–5.233)	5.47 (2.087–8.179)

### 3.2 Survival Rate

**FIGURE 1:**
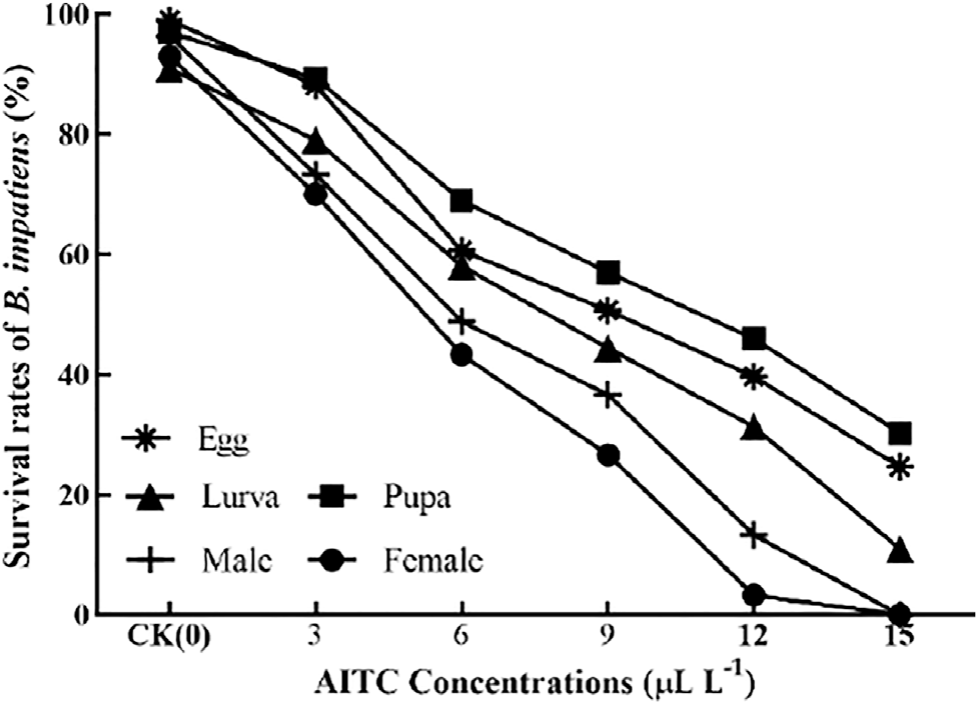
Effect of different concentrations of AITC on the survival rate of *B. impatiens*.

The bioassay results showed that increasing concentrations of AITC significantly (*p* < 0.05) decreased the survival rate of *B. impatiens* each stage (Figure 1), that is, significantly increased their mortality rates. Fumigation results showed that AITC had suppressive effects on each stage of *B. impatien*. For example, at 6 μL/L concentration of AITC, the survival rate of pupae (69%) was the least affected by AITC, with the highest survival rate and the strongest resistance, followed by eggs (60%) and larvae (58%). A concentration of 15 μL/L AITC was fatal to female and male adults, which caused 100% mortality.

### 3.3 Larval Stage and Pupal Stage

**FIGURE 2:**
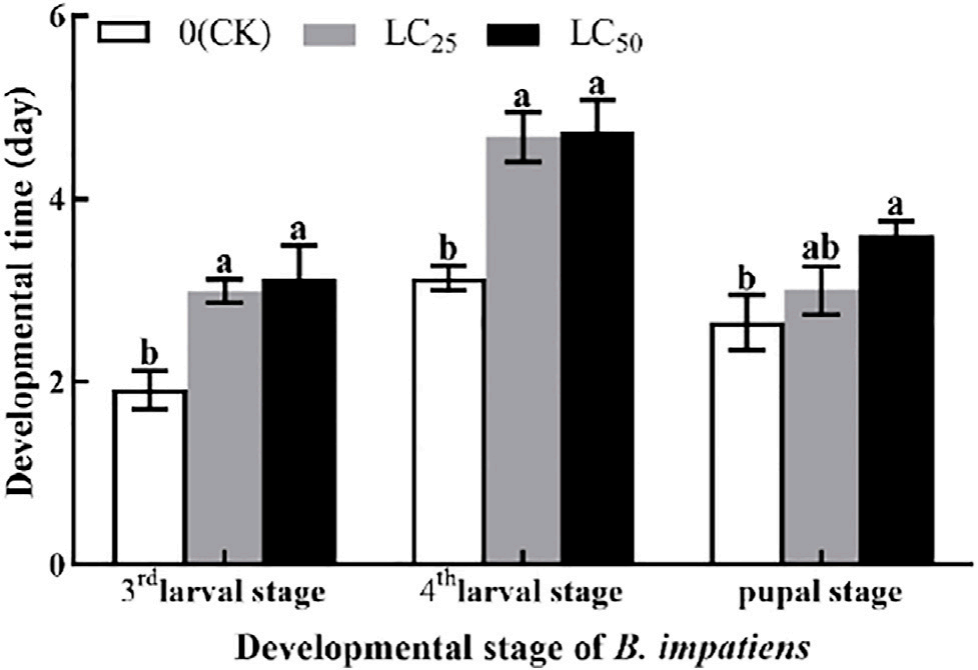
Changes in the third larval stage, fourth larval stage, and pupal stage of *B. impatiens* after sub-lethal concentration fumigation of AITC. Data represent mean ± SD of three replicates. Different lower-case letters indicate statistically significant difference between sub-lethal concentrations by Tukey’s test (*p* < 0.05).

The developmental stage of *B. impatiens* was significantly (*p* < 0.05) affected by LC_25_ and LC_50_ fumigation treatment (Figure 2). The third larval stage was significantly (*p* < 0.05) prolonged after LC_25_ and LC_50_ of AITC stress; however, no obvious changes between those two sub-lethal concentrations were observed. AITC stress of LC_25_ prolonged third larval stage of 0.77 d, compared with LC_50_. After AITC treatment, LC_50_ prolonged the fourth larval stage by 0.05 d than LC_25,_ compared with CK (3.13 d). The fumigation treatment prolonged the pupal stage of *B. impatiens* by 0.35 d at LC_25_ and 0.95 d at LC_50_. However, there was no significant difference between CK and LC_25_.

### 3.4 Pupation Rate, Pupal Weight, and Adult Emergency Rate

**FIGURE 3:**
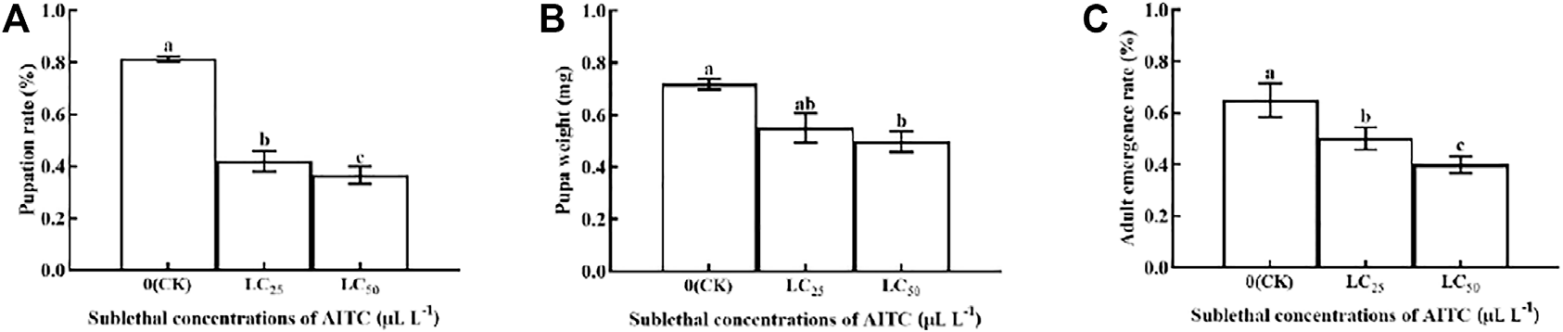
Changes in the pupation rate **(A)**, pupa weight **(B),** and adult emergence rate **(C)** of *B. impatiens* after sub-lethal concentration fumigation of AITC. Data represent mean ± SD of three replicates. Different lower-case letters indicate statistically significant difference between sub-lethal concentrations by Tukey’s test (*p* < 0.05).

The pupation rate, pupal weight, and adult emergency rate were significantly (*p* < 0.05) affected by AITC fumigation. The pupation rate was the lowest after fumigation treatment of AITC at LC_50_ (36.67%), followed by LC_25_ (41.94%), compared with the CK (81.39%) (Figure 3A). Pupal weight was reduced by 0.10 mg at LC_25_ and by 0.22 at LC_50_; however, there was no significant difference between the two sub-lethal concentrations (Figure 3B). The adult emergency rate in CK was significantly higher (65%; *p* < 0.05) than that of LC_25_ (50.17%) and LC_50_ (40%) treatments (Figure 3C).

### 3.5 Adult Longevity and Oviposition

**FIGURE 4:**
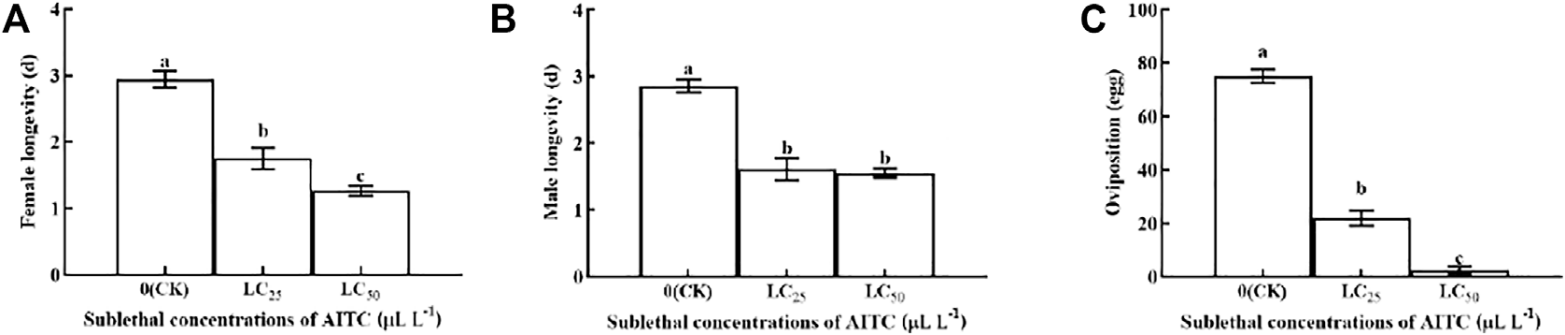
Changes in female longevity **(A)**, male lingevity **(B),** and adult oviposition **(C)** of *B. impatiens* after sub-lethal concentration fumigation of AITC. Data represent mean ± SD of three replicates. Different lower-case letters indicate statistically significant difference between sub-lethal concentrations by Tukey’s test (*p* < 0.05).

Female longevity was significantly (*p* < 0.05) shortened by fumigation at LC_25_ (1.75 d) and LC_50_ (1.64 d), compared with that in CK (2.94 d; Figure 4A). Male longevity was shorter at LC_25_ (1.56 d) than at LC_50_ (1.25 d) and had no significant difference between the two treatments (Figure 4B). Oviposition was significantly (*p* < 0.05) decreased at LC_25_ (22 eggs) and at LC_50_ (0 eggs) compared to the CK (75.10 eggs; Figure 4C). Obviously, AITC fumigation treatment was significantly inhibited by the oviposition of *B. impatiens*.

### 3.6 Allyl Isothiocyanate Fumigation Efficiency Under High CO_2_

**FIGURE 5:**
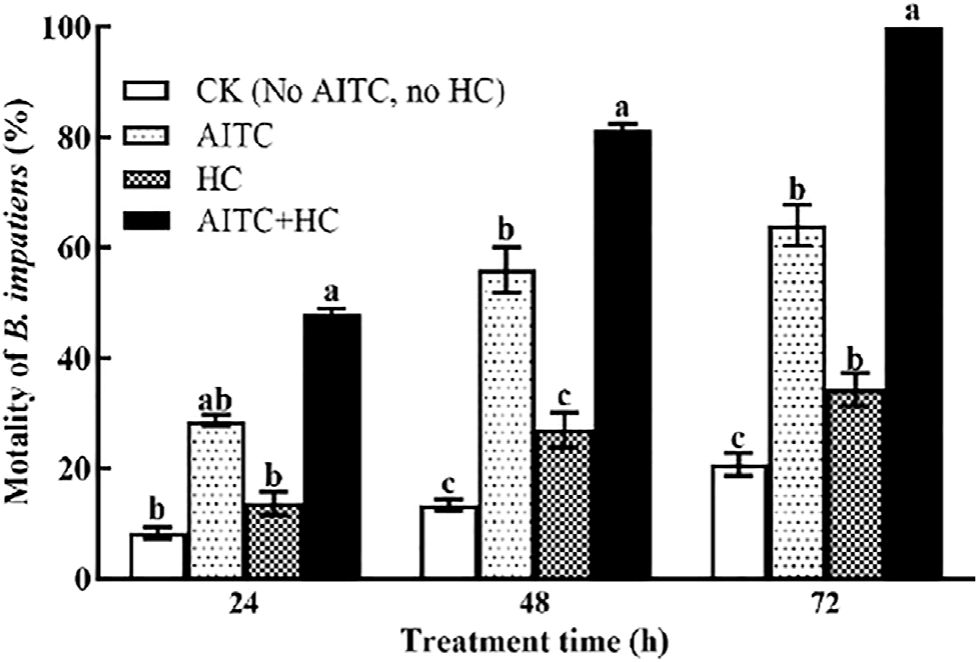
Changes in mortality of *B. impatiens* in three treatment hours under ambient CO_2_ or/and high CO_2_ conditions with and without AITC fumigation. CK: no AITC fumigation under ambient CO_2_ condition; AF: AITC fumigation; HC: high CO_2_ stress; AF + HC: AITC fumigation under high CO_2_ condition. Data represent mean ± SD of three replicates. Different lower-case letters indicate statistically significant difference between sub-lethal concentrations by Tukey’s test (*p* < 0.05).

To determine the effect of high CO_2_ on AITC fumigation efficiency against *B. impatiens*, we incorporated high CO_2_ exposure of the third larvae during AITC LC_25_ treatment (Figure 5). Notably, high CO_2_ and/or AITC treatment apparently enhanced the mortality of *B. impatiens* (*p* < 0.05) with prolonged time. The larvae subjected to the AITC treatment alone displayed 28.67, 39, and 83.67% mortality at 24, 48, and 72 h time points, respectively. Exposure to high concentration of CO_2_ during AITC treatment substantially increased the mortality to 48, 59.33, and 99%.

### 3.7 Detoxification Enzyme Activity

**FIGURE 6:**
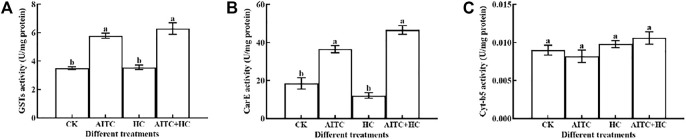
Changes in GST enzyme activity **(A)**, CarE enzyme activity **(B),** and Cyt-b5 enzyme **(C)** activity of *B. impatiens* after AITC fumigation treatment under ambient CO_2_ or high CO_2_ conditions with and without AITC fumigation. CK: no AITC fumigation under ambient CO_2_ condition; AF: AITC fumigation; HC: high CO_2_ stress; AF + HC: AITC fumigation under high CO_2_ condition. Data represent mean ± SD of three replicates. Different lower-case letters indicate statistically significant difference between sub-lethal concentrations by Tukey’s test (*p* < 0.05).

Generally, AITC fumigation treatments had a significant (*p* < 0.05) effect on the detoxification enzyme activities. We measured GST, CraE, and Cyt-b5 activities in third larvae of *B. impatiens* after high CO_2_ and/or AITC fumigation for 24 h followed by recovery of normal environment for 2 h (Figure 6). GST activity was upregulated by either the AITC treatment alone or the combined treatment of AITC with high CO_2_, and the latter induced a higher response (Figure 6A). Similarly, CarE activity was also increased by these treatments (Figure 6B). Substantially higher CarE activity was obtained after the combined treatment than after individual treatment in third larvae. Enhanced Cyt-b5 activity was also detected under various treatments compared to that in the untreated group (Figure 6C), and high CO_2_ apparently stimulated these responses than being AITC used alone. Elevated detoxification enzymes may reduce the damage caused by AITC.

### 3.8 Antioxidant Enzyme Activity

**FIGURE 7:**
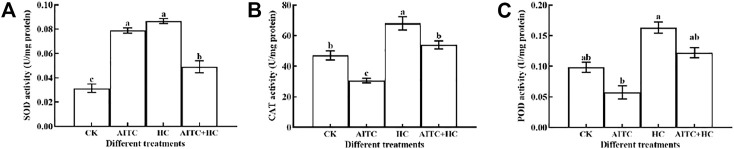
Changes in SOD enzyme activity **(A)**, CAT enzyme activity **(B)**, and POD enzyme activity **(C)** of *B. impatiens* after AITC fumigation treatment under ambient CO_2_ or high CO_2_ conditions with and without AITC fumigation. CK: no AITC fumigation under ambient CO_2_ condition; AF: AITC fumigation; HC: high CO_2_ stress; AF + HC: AITC fumigation under high CO_2_ condition. Data represent mean ± SD of three replicates. Different lower-case letters indicate statistically significant difference between sub-lethal concentrations by Tukey’s test (*p* < 0.05).

High concentration of CO_2_ often corresponds to low concentration of O_2_. This environment will stimulate the ROS of organisms and produce oxygen oxidative damage. After being fumigated by AITC and recovered indoors for 2 h, the activity of antioxidant enzymes *in vivo* was detected. The results showed that the activities of CAT and POD were inhibited, while the activities of SOD were significantly induced (*p* < 0.05; Figure 7). Under high CO_2_ concentration condition, the activities of SOD, CAT, and POD were significantly increased (*p* < 0.05). The activity of SOD significantly stimulated when treated by AITC under high CO_2_ compared with that of CK; however, the degree of activation was weaker than that of the two separate treatments (Figure 7A).

## 4 Discussion

Plant extracts are considered for pest management because they can be selective, biodegraded to non-toxic products, and have low toxicity to non-target organisms and environment (Ren et al., 2018). Allyl isothiocyanate (AITC) is a natural product formed from allyl glucosinolate hydrolysis and obtained from the damaged tissues of cruciferous plants (Clarke, 2010; Williams et al., 2015). Shi et al. (2017) reported that AITC has high fumigation bioactivity against eggs, larvae, pupae, and adults of *B. odoriphag*a (Shi et al., 2017). The application of AITC inhibits *S. zeamai* performance, particularly at the adult stage (Wu H. et al., 2014). In the present study, the AITC fumigation test showed greater lethal effects on female and male adults of *B. impatiens* than those on eggs, third larvae, and pupae. This is probably because the AITC fumigation contains high toxicity that might have interfered with their respiratory mechanism. We, therefore, speculate that AITC fumigation is more lethal to the adult *B. impatiens* and sub-lethal to its eggs, third larvae, and pupae stages. Consequently, the life cycle of *B. impatiens* should be considered when developing a pesticide application strategy, as its eggs, third larvae, and pupae stages are spent in the growing medium than on the host plant.

In indoor or outdoor field environment, insecticides produce lethal and sub-lethal effects on pests (Leviticus et al., 2020). Sub-lethal effects refer to ecological, physiological, or behavioral changes in surviving insects after being exposed to sub-lethal insecticide concentrations, which cannot kill them immediately but inhibits their growth, reproduction, and longevity (Li Z et al., 2018; Wang Q et al., 2019). The third larvae of *Helicoverpa armigera* when treated with LC_10_, LC_25,_ and LC_50_ of chlorantraniliprole for 48 h showed significantly prolonged larval and pupal stages of the first generation (Ou et al., 2012). Liang et al. (2017) reported that the growth, development, and fecundity of *Spodoptera litura* third larvae were significantly inhibited by thiacloprid LC_25_ and LC_50_ (Liang et al., 2017). To understand toxicity impact for AITC on *B. impatiens*, its lethal and sub-lethal effects on third larvae were investigated. The results showed that larval and pupal stages were prolonged, pupae weight was lightened, rates of pupation and adult development were reduced, and oviposition was decreased after the third larvae were treated with LC_25_ and LC_50_ of AITC.

It is well-recognized that CO_2_, as a regulator of respiration, plays very important roles in the life activities of insects (Miller, 1974). However, when the CO_2_ concentration increased by 10–20%, insect spiracles remained open permanently (Miller, 1974), which provided a new idea for the efficient utilization of the fumigant. High CO_2_ combined with fumigation against many storage pests has been well-reported. Leelaja et al. (2007) found that elevated CO_2,_ approximately 10–20%, could significantly enhance the fumigation effect of allyl acetate on *S. oryzae*, *S. serrulata*, *T. ferrugineus*, *T. castaneum*, and *S. Dominica* (Leelaja et al., 2007). Similarly, high CO_2_ increased the effectiveness of methyl benzoate against the larvae of *Callosobruchus chinensis* (Wang L et al., 2019) and significantly improved ethyl formate toxicity to *T. castanea* and *Rhizopertha dominica* (Haritos et al., 2006). Our findings illustrated that the exposed third larvae of *B. impatiens* to AITC under high CO_2_ conditions lead to a higher mortality than in normal atmosphere; moreover, these lethal effects were as high as 100% with prolonged time. This indicates that high CO_2_ concentration probably contributed greatly to the fumigant formation, which caused a high lethal effect on *B. impatiens*. Accordingly, the presence of CO_2_ concentration under green and mushroom houses must be examined when considering the management of *B. impatiens.*

Insects detoxify the different numerous exogenous and endogenous compounds due to the production of detoxification enzymes, including GSTs, CarE, and Cyt-b5, and their activities are considered to be an effective indicator monitoring the development of insect resistance to pesticides (Li et al., 2021; Bilal et al., 2020). The presence of insecticides is reported to stimulate the activities of detoxification enzymes due to the increased production of ROS (Jiang et al., 2020). The present results show that the enzyme activities of GSTs, CarE, and Cyt-b5 in *B. impatiens* third larvae were induced by the sub-lethal concentrations of AITC (LC_25_) after 24 h and significantly enhanced under high CO_2_. These results suggest that the detoxification enzymes may be adaptively activated in *B. impatiens* third larvae as a pro-survival mechanism in response to low concentration of AITC (LC_25_). Although *B. impatiens* developed resistance to some extent, it was not enough to defend them from the lethal effect of the AITC (LC_25_) under high CO_2_. Previous studies reported that increase in AITC exposure could influence the host plant physiology through accumulation of ROS. Although the present study speculates that AITC fumigation shows greater sub-lethal effects on the growth and reproduction of *B. impatiens,* AITC under high CO_2_ conditions may lead to host plant cell damage due to high accumulation of ROS. Thus, the level and frequency of AITC fumigation application must be considered in pest management.

## 5 Conclusion

The results of this study indicate that AITC is an effective plant extract which demonstrates greater lethal effects on the various stages of *B. impatiens*. The fumigation method showed effective insecticidal effect on the *B. impatiens*. AITC (LC_25_ and LC_50_) greatly showed sub-lethal effects on the growth and reproduction of *B. impatiens*. The AITC fumigation test showed greater lethal effects on female and male adults of *B. impatiens* than those on eggs, third larvae, and pupae. LC_25_ and LC_50_ of AITC prolonged larval and pupal stages. The rates of pupation and adult development were reduced, and oviposition was decreased after third larvae were under AITC stress. Moreover, high CO_2_ enhanced the insecticidal effects of AITC and caused changes in detoxification and antioxidant enzyme activities in *B. impatiens*. The fumigation method in the application of AITC can be useful in areas where *B. impatiens* is a major concern. Although the present study speculates that AITC fumigation shows greater sub-lethal effects on the growth and reproduction of the *B. impatiens,* AITC under high CO_2_ conditions may lead to host plant cell damage due to high accumulation of ROS. Thus, the level and frequency of AITC fumigation application must be considered in pest management.

## Data Availability

The original contributions presented in the study are included in the article/Supplementary Materials; further inquiries can be directed to the corresponding author.
